# Phenylpyruvate Contributes to the Synthesis of Fragrant Benzenoid–Phenylpropanoids in *Petunia* × *hybrida* Flowers

**DOI:** 10.3389/fpls.2017.00769

**Published:** 2017-05-12

**Authors:** Moran Oliva, Einat Bar, Rinat Ovadia, Avichai Perl, Gad Galili, Efraim Lewinsohn, Michal Oren-Shamir

**Affiliations:** ^1^Department of Ornamental Plants and Agricultural Biotechnology, Agricultural Research Organization, Volcani CenterRishon LeZion, Israel; ^2^Department of Plant Sciences, The Weizmann Institute of ScienceRehovot, Israel; ^3^Department of Vegetable Crops, Newe Ya’ar Research Center, Agricultural Research OrganizationRamat Yishay, Israel; ^4^Department of Fruit Tree Science, Agricultural Research Organization, Volcani CenterRishon LeZion, Israel

**Keywords:** benzenoid–phenylpropanoid, fragrant volatiles, phenylpyruvate, *PheA^∗^*, specialized metabolism

## Abstract

Phenylalanine (Phe) is a precursor for a large group of plant specialized metabolites, including the fragrant volatile benzenoid–phenylpropanoids (BPs). In plants, the main pathway leading to production of Phe is *via* arogenate, while the pathway *via* phenylpyruvate (PPY) is considered merely an alternative route. Unlike plants, in most microorganisms the only pathway leading to the synthesis of Phe is *via* PPY. Here we studied the effect of increased PPY production in petunia on the formation of BPs volatiles and other specialized metabolites originating from Phe both in flowers and leaves. Stimulation of the pathway *via* PPY was achieved by transforming petunia with *PheA^∗^*, a gene encoding a bacterial feedback insensitive bi-functional chorismate mutase/prephenate dehydratase enzyme. *PheA^∗^* overexpression caused dramatic increase in the levels of flower BP volatiles such as phenylacetaldehyde, benzaldehyde, benzyl acetate, vanillin, and eugenol. All three BP pathways characterized in petunia flowers were stimulated in *PheA^∗^* flowers. In contrast, *PheA^∗^* overexpression had only a minor effect on the levels of amino acids and non-volatile metabolites both in the leaves and flowers. The one exception is a dramatic increase in the level of rosmarinate, a conjugate between Phe-derived caffeate and Tyr-derived 3,4-dihydroxyphenylacetate, in PheA^∗^ leaves. *PheA^∗^* petunia flowers may serve as an excellent system for revealing the role of PPY in the production of BPs, including possible routes directly converting PPY to the fragrant volatiles. This study emphasizes the potential of the PPY route in achieving fragrance enhancement in flowering plants.

## Introduction

**FIGURE 1:**
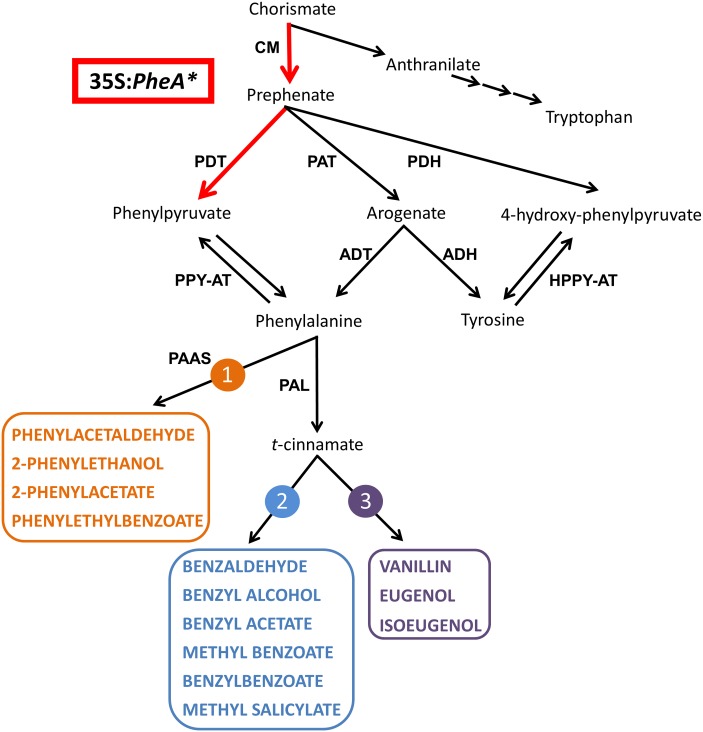
**A schematic diagram of the AAA and BP metabolic networks in petunia flowers**. The enzymatic steps performed by the bi-functional feedback insensitive PheA^∗^ enzyme are marked in red. Enzymes are marked in bold capital letters. The volatile compounds are marked by colored bold capital letters. Multiple arrows mark several biochemical reactions. The three BP volatile metabolic pathways originating from Phe in petunia flowers are marked in orange **(1)**, blue **(2)**, and purple **(3)**. ADH, arogenate dehydrogenase; ADT, arogenate dehydratase; CM, chorismate mutase; HPPY-AT, hydroxyphenylpyruvate aminotransferase; PAAS, phenylacetaldehyde synthase; PAL, phenylalanine ammonia lyase; PAT, prephenate aminotransferases; PDH, prephenate dehydrogenase; PDT, prephenate dehydratase; PPY-AT, phenylpyruvate aminotransferase.

The fragrant volatile benzenoid–phenylpropanoids (BPs) are part of a large group of plant specialized metabolites originating from the aromatic amino acid (AAA) phenylalanine (Phe). Additional metabolites originating from Phe include flavonoids, anthocyanin pigments, as well as phenolic soluble metabolites with anti-microbial and anti-oxidant characteristics (Vogt, 2010; Dudareva et al., 2013). The shikimate pathway serves as a bridge between primary metabolism and the biosynthesis of AAAs and specialized metabolites derived from them (Tzin and Galili, 2010). The linking metabolite between the shikimate and AAA pathways is chorismate (**Figure 1**). Chorismate is also a branching point between the Trp and Tyr-Phe biosynthetic pathways suggesting that it may be a regulatory point in the synthesis of each. In plants, most of the carbon flux originating from the shikimate pathway is directed toward the synthesis of Phe and its downstream phenylpropanoids (Tzin and Galili, 2010; Yoo et al., 2013).

The Phe-Tyr biosynthesis pathways have been studied extensively in recent years and were shown to be tightly connected and inter-regulated (Maeda and Dudareva, 2012; Yoo et al., 2013; Schenck et al., 2015). In petunia, they begin with the transition of chorismate to prephenate (**Figure 1**) and continue through several parallel pathways to generate Phe and Tyr. Two of the parallel pathways share arogenate as an intermediate that is then transformed either into Phe or Tyr. Two additional pathways are *via* 4-hydroxyphenylpyruvate (4-HPPY) for the formation of Tyr, and *via* phenylpyruvate (PPY) for the formation of Phe (**Figure 1**). In addition to being a precursor to Phe, PPY has also been shown to be a Phe catabolic product and precursor for BP volatiles, as was shown in rose flowers (Watanabe et al., 2002; Hirata et al., 2012).

In plants, the main pathway leading to production of Phe is *via* arogenate, while the PPY-Phe pathway is considered merely an alternative route. Several studies in plants present evidence showing that the PPY-Phe is in fact active. For example, etiolated *Arabidopsis* seedlings respond to light by enhancement of PPY levels in the cytosol, leading to enhanced Phe biosynthesis (Warpeha et al., 2006). Another example is induction of the PPY-Phe route following inhibition of the arogenate-Phe pathway both in *Petunia* × *hybrida* flowers (Yoo et al., 2013) and *Nicotiana benthamiana* leaves (de la Torre et al., 2014). Furthermore, in petunia flowers high flux through the shikimate pathway, either by feeding experiments (Maeda et al., 2010) or by metabolic engineering (Oliva et al., 2015), activated the PPY-Phe route.r

Unlike plants, in most microorganisms the only pathway leading to the synthesis of Phe is *via* PPY (Prabhu and Hudson, 2010; Yoo et al., 2013). In one family of bacteria (Enterobacteriaceae), a bi-functional enzyme, PheA, converts chorismate *via* prephenate to PPY (Zhang et al., 1998; Tzin et al., 2009). In *Arabidopsis* seedlings, overexpression of the bacterial PheA enzyme in a feedback insensitive form (PheA^∗^) led to the accumulation of Phe and Phe-derived metabolites (Tzin et al., 2009).

Petunia serves as a model for studying the regulation of AAA biosynthesis (Colquhoun et al., 2010; Maeda et al., 2011) and for the biosynthesis and regulation of AAA-derived metabolites, including volatile BPs (Colquhoun and Clark, 2011; Sheehan et al., 2012; Dudareva et al., 2013; Muhlemann et al., 2014). Recently, we showed that overproduction of the AAAs in the purple petunia (cv. V26) resulted in increased levels of Phe-derived volatiles, but had no effect on anthocyanin pigmentation (Oliva et al., 2015).

The major group of volatiles synthesized by petunia flowers is BPs, with lesser amounts of terpenoids and short-chain fatty acids (Pichersky et al., 2006; Dudareva et al., 2013; Muhlemann et al., 2014). The BP volatiles of petunia include biosynthesis of several structural backbones of either benzenoid (C6–C1) or phenylpropanoid (C6–C2/3), synthesized *via* three parallel metabolic pathways. One is derived directly from Phe, and the other two branching from *t*-cinnamate or *p*-coumarate (Muhlemann et al., 2014; **Figure 1**).

In the formation of BPs, Phe serves as a substrate for the two enzymes, phenylacetaldehyde synthase leading to pathway no. 1 and Phe ammonia lyase (PAL), leading to pathways 2 and 3 (**Figure 1**; Vogt, 2010; Muhlemann et al., 2014). Both in *Arabidopsis* and petunia, PPY was shown to serve as a Phe precursor (Tzin et al., 2009; Yoo et al., 2013). However, in rose flowers, it was shown that the reverse reaction can occur, converting Phe to PPY, which is then converted into 2-phenylethanol *via* several BP pathways (Watanabe et al., 2002; Hirata et al., 2012, 2016). A similar observation was obtained in melon fruit tissue fed with labeled Phe (Gonda et al., 2010). Recently, the aminotransferase *Ph*PPY-AT was identified in petunia and was shown to have higher affinity to Phe production from PPY in comparison to the reverse reaction (Yoo et al., 2013).

The pivotal role of Phe in synthesizing fragrant BPs, anthocyanins, and phenolic soluble metabolites *via* the arogenate pathway in petunia flowers has been studied extensively. However, there is only scarce evidence for the role of the PPY pathway in the biosynthesis of volatiles and pigmented molecules. Here, we focused on the effect of directing carbon flux toward PPY, bypassing the arogenate pathway, on the production of specialized metabolites, and in particular BPs. This was done by overexpressing the bacterial feedback insensitive *PheA^∗^* gene in the purple petunia cultivar V26, and following its effect on the specialized metabolic profile of the leaves and flowers. *PheA*^∗^ flowers accumulated significantly high levels of fragrant BP volatiles, emphasizing the potential of the PPY route, normally of minor importance in plants, in enhancing fragrance production.

## Materials and Methods

### Plant Material, Growth, and Tissue Sampling

Petunia (*Petunia* × *hybrida*) Vilm V26 wild-type (WT) cultivar (Napoli and Ruehle, 1996) was used to generate transgenic *PheA^∗^* and control plants. WT and transgenic plants were grown as described in Oliva et al. (2015).

Leaves were collected from plants before flowering. Petals were collected, if not mentioned otherwise, 1–2 days post-anthesis, at mid-day (12:00–14:00). When night analysis was performed, samples were collected at midnight (23:00–24:00). Samples for metabolomics profiling were frozen in liquid nitrogen and stored at -80°C.

### Stable Transformation

Sterile petunia plants were grown from surface sterilized seed on solidified MS medium (Murashige and Skoog, 1962) supplemented with 3% (w/v) sucrose. Transformation was performed as published earlier (Oliva et al., 2015).

### Plasmid Construction, Transgene Validation by Immunoblot Analysis

The *PheA^∗^* construct, which was used for generating transgenic petunia *PheA*^∗^ plants, was described previously (Tzin et al., 2009). It contains a 35S CaMV promoter, a Rubisco small subunit-3A plastid transit peptide (TP) fused in frame to 5′ end of the truncated *PheA* feedback insensitive bi-functional bacterial gene. The *PheA^∗^* construct lacks the regulatory C-terminal domain, making the enzyme feedback insensitive to Phe. Three copies of the hemagglutinin (HA) epitope tag were fused to the 3′ end of *PheA*^∗^ for tracking the protein. For controls, WT plants were transformed with the same plasmid used for transformation, containing only the kanamycin resistance cassette. *PheA*^∗^ construct was introduced into *Agrobacterium tumefaciens* strain EHA-105.

To determine the levels of the transgenic enzyme and to select lines for characterization, immunoblots were performed as previously described (Stepansky and Galili, 2003), using monoclonal anti-HA antibodies (Santa Cruz Biotechnology Inc, Dallas, TX, USA) with the following modifications. For leaf protein extraction, the ratio of tissue weight/protein extraction buffer (PEB) was 1 g:1 v, while for petal protein extraction, the ratio was 1 g:3 v. Protein quantification was determined using the Pierce © bicinchoninic acid (BCA) protein assay kit (Thermo Fisher Scientific, USA).

### GC-MS Extraction, Derivatization, and Profiling of Polar Soluble, Non-volatile Extracts

Samples (*n* = 3) of leaves and petals of the petunia *PheA^∗^* and control plants were used for analysis of the semi-polar and polar soluble metabolites by gas chromatography-mass spectrometry (GC-MS), as previously described (Tzin et al., 2012; Oliva et al., 2015). The data was analyzed using Xcalibur software v.1.4 (Thermo Finnigan), and the compounds were identified by comparison of their retention index (RI) and mass spectrum to those generated by authentic standards analyzed using the same column and similar conditions. If standards were not available, compounds were putatively identified by comparison of their RI and mass spectrum to those present in the mass spectra GMD VAR5 library of Max-Planck-Institute for Plant Physiology, Golm, Germany, and the commercial mass spectra library NIST05^1^. The response values for metabolites resulting from the Xcalibur processing method were normalized to the ribitol internal standard.

### Statistics

Statistical significance was determined either by a *t*-test or by one-way ANOVA test, using JMP software or by Metaboanalyst^2^ (Xia et al., 2009; Xia and Wishart, 2011). Hierarchical clustering was performed on absolute levels of volatile metabolites using Expander software (Ulitsky et al., 2010).

### GC-MS Analysis of Volatiles Metabolites

Petunia corollas were harvested for analysis of internal pools volatiles at 1–2 days post-anthesis at mid-day (12:00–14:00), to minimize possible effects of rhythmicity, frozen and ground to powder. Upon analysis, 100–200 mg of powdered sample were added into a 10 ml glass vial [DuPont autosampler vial (DuPont Performance Elastomers) with a solid-top polypropylene cap (Alltech)] containing 1 ml of 20% (w/v) NaCl solution (to stop enzymatic activity), 0.3 μg of 2-heptanone (as an internal standard) and 5 mM of sodium meta bisulfide, to avoid sample browning. The vial was sealed and stored at room temperature to reach equilibrium of the gas phase, for 12–24 h until analyzed. Solid-phase micro extraction (SPME) sampling was conducted according to Davidovich-Rikanati et al. (2008), with minor modifications. A 65 μm fused silica fiber coated with polydimethylsiloxane/divinylbenzene (PDMS/DVB) (Supelco Inc) was used. Samples were pre-heated to 50°C, agitated for 5 min at 500 rpm, before insertion of fiber and exposure to the sample headspace. After 25 min the SPME syringe was introduced into the injector port of the GC-MS apparatus for further analysis. Volatile compounds were separated on a GC-MS apparatus (Agilent Technologies) equipped with an Rtx-5 SIL MS (30 m × 30.25 mm × 30.25 μm) fused-silica capillary column (Restek Co.). Helium was used as a carrier gas (0.8 ml/min). The injector temperature was 250°C, set for splitless injection. The oven was set to 50°C for 1 min, and then the temperature was increased to 180°C at a rate of 5°C/min, then to 260°C at 20°C/min. Thermal desorption was allowed for 10 min. The detector temperature was set at 280°C. The mass range was recorded from 41 to 250 *m/z*, with electron energy of 70 eV. A mixture of straight-chain alkanes (C7–C23) was injected into the column under the aforementioned conditions for determination of retention times. The identification and quantification of the volatiles was done according to Davidovich-Rikanati et al. (2008), with the exception that the area used in the calculation for quantification, was of the major peak area, multiplied by a correction factor ratio.

### Volatiles’ Annotation Using MSDchem

Compounds were identified by comparison of their retention times and mass spectra to both their retention index (RI) and mass spectra of authentic standards. When no authentic standards were available, RI and mass spectra were compared with MS libraries. The levels of phenethyl acetate, phenylethyl alcohol, eugenol, benzyl alcohol, benzeneacetaldehyde, and benzaldehyde were quantified using calibration curves obtained from authentic standards.

### Anthocyanins and Flavonoids Extraction and Separation by HPLC

Petunia petals were collected at 1–2 days post-anthesis at mid-day (0.1–0.3 g) and frozen in liquid nitrogen. Procedure for anthocyanin extraction and analysis in HPLC was performed according to Oliva et al. (2015).

## Results

### Generation of Transgenic PheA^∗^ Petunia Lines and Metabolic Characterization of Their Leaves

*Petunia* × *hybrida* V26 plants were transformed with the bacterial feedback insensitive bi-functional chimeric *PheA* gene (marked as *PheA^∗^*) under the 35S promoter, targeted to the chloroplast by a TP (Tzin et al., 2009). This bacterial chimeric PheA^∗^ enzyme includes two functional domains, encoding for two enzymatic activities in the AAA biosynthesis pathway. The N-terminal domain encodes for a chorismate mutase (CM) activity, catalyzing the transition from chorismate to prephenate, and the C-terminus encodes for the prephenate dehydratase (PDT) activity, catalyzing the transition from prephenate to PPY (**Figure 1**). The PheA^∗^ enzyme was engineered so that it lacks the regulatory C-terminal domain causing it to be feedback insensitive and enabling direction of the carbon flux toward PPY (Tzin et al., 2009).

**FIGURE 2:**
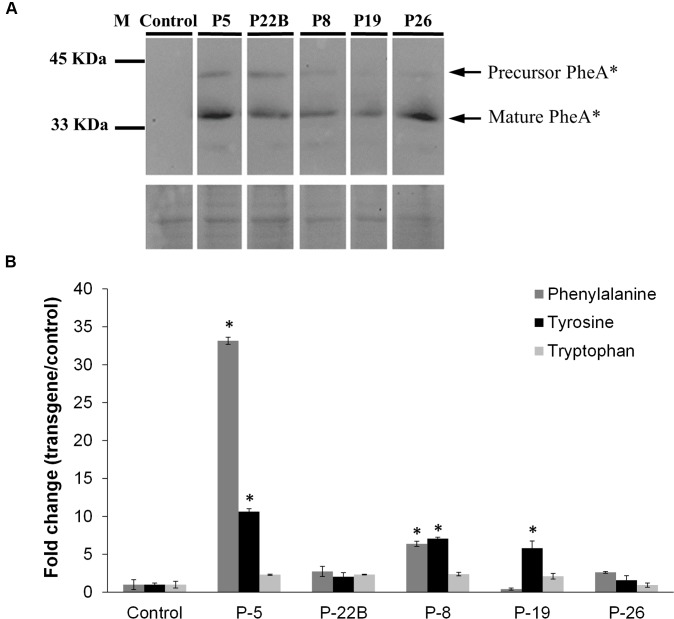
**Effect of PheA^∗^ protein abundance on the AAAs and shikimate pathway intermediates in the leaves. (A)** Accumulation of PheA^∗^ protein in the leaves of the five transgenic plants. Immunoblot analysis was performed using anti-HA antibody (1:1000). Lower panel indicates similar protein loading by Amido-black staining. **(B)** AAA levels in the leaves of the *PheA^∗^* lines. Results are presented as fold change enhancement (*n* = 3). Asterisks indicate *p* ≤ 0.05 statistically significant difference between the *PheA^∗^* line and control, using ANOVA followed by Dunnett’s *post hoc* test. Bars on top of the histograms indicate standard errors.

Five independently transformed petunia lines were selected for metabolic analysis. Two forms of the PheA^∗^ polypeptide, including the immature form (∼40 kDa) and cleaved mature form (34 kDa) were detected by the HA antibodies (**Figure 2A** and **Supplementary Figure S1**). These results were similar to previously observed results in *Arabidopsis PheA^∗^* leaves (Tzin et al., 2009).

Leaves from the five selected lines and from control plants (transgenic plants expressing only the kanamycin resistance gene) were subjected to GC-MS analysis to detect metabolites related to the AAA pathway that accumulate in the transgenic lines. There was no clear correlation between Phe levels and PheA^∗^ levels in the five transgenic lines: the highest Phe level was in line 5, with a relatively high level of PheA^∗^. However, the second line with a significantly higher level of Phe was line 8, with a relatively low level of PheA^∗^. Tyr levels were significantly higher in three of the five transgenic lines, up to 10-fold higher in line 5. Here too, Line 26 had a relatively high level of PheA^∗^ with no significant increase both in Phe and Tyr levels (**Figure 2B**). As expected, Trp levels remained unaltered in the *PheA^∗^* lines (**Figure 2B**).

Several AAA precursors along the shikimate and AAA pathways accumulated to higher levels in *PheA^∗^* leaves, in comparison to the control (**Table 1**). 4-HPPY was higher in lines 8 and 19. PPY, the direct product of PheA^∗^ enzyme, accumulated significantly in leaves of *PheA*^∗^ 5. 3-Phenyllactate, synthesized directly from PPY (Watanabe et al., 2002; Bedewitz et al., 2014; **Figure 6**) accumulated to significantly higher levels in the transgenic lines 5 and 8 (**Table 1**).

**Table 1:** Effect of *PheA^∗^* protein abundance on metabolites derived from the shikimate, AAA, and AAA-derived pathways in leaves.

Compound name	Control	P-5	P-22B	P-8	P-19	P-26
Benzoate	1 ± 0.28	**7.30 ± 0.76**	0.78 ± 0.1	2.31 ± 0.16	1.24 ± 0.03	0.93 ± 0.01
*t*-Caffeate	1 ± 0.61	2.32 ± 0.21	4.00 ± 0.62	**12.90 ± 0.12**	**6.87** ±**0.24**	**5.98 ± 0.5**
*t*-Coumarate	1 ± 0.77	1.83 ± 0.19	3.43 ± 0.78	**5.68 ± 0.33**	1.06 ± 0.19	1.05 ± 0.46
Coniferyl alcohol	1 ± 0.72	0.51 ± 0.36	**3.36 ± 0.47**	1.13 ± 0.29	2.87 ± 0.55	2.63 ± 0.36
Ferulate	1 ± 0.52	**2.89 ± 0.33**	1.18 ± 0.48	2.23 ± 0.61	**9.13 ± 0.89**	1.01 ± 0.5
3-Hydroxybenzoate	1 ± 0.59	1.51 ± 0.21	1.21 ± 0.4	2.57 ± 0.21	1.09 ± 0.24	1.15 ± 0.36
4-Hydroxybenzoate	1 ± 0.44	0.67 ± 0.3	1.04 ± 0.22	1.91 ± 0.09	1.39 ± 0.12	1.18 ± 0.1
4-Hydroxy-3-methoxyphenylethylene glucopyranoside	1 ± 0.31	0.93 ± 0.51	1.16 ± 0.33	1.70 ± 0.38	1.20 ± 0.18	1.31 ± 0.15
4-Hydroxyphenyl-β-glucopyranoside	1 ±0.42	**4.27 ± 0.08**	1.35 ± 0.18	**14.13 ± 0.19**	**2.92 ± 0.13**	1.97 ± 0.26
4-Hydroxyphenylpyruvate	1 ± 0.71	0.77 ± 0.07	0.26 ± 0.35	**8.13 ± 0.19**	**6.01 ± 0.31**	0.26 ± 0.47
3-Phenyllactate	1 ± 0.40	**10.8 ± 0.30**	1.64 ± 0.21	**5.37 ± 0.51**	2.40 ± 0.07	1.64 ± 0.43
Phenylpyruvate	1 ± 0.45	**2.7 ± 0.06**	1.09 ± 0.30	2.08 ± 0.20	1.39 ± 0.16	0.89 ± 0.42
Rosmarinate	1 ± 0.07	**10.49 ± 0.43**	1.25 ± 0.61	**818.80 ± 0.4**	**302.5 ± 0.72**	2.27 ± 0.71
Salicylic acid-glucopyranoside	1 ± 0.28	2.52 ± 0.43	2.00 ± 0.34	**4.08 ± 0.23**	**4.81 ± 0.37**	2.15 ± 0.35

*Results are presented as fold change of transgene/control ± SE (*n* = 3). Statistical significant results are marked by bold. Analysis was performed by ANOVA followed by Dunnett’s *post hoc* test with *p* ≤ 0.05 following log transformation of the absolute results*.

To evaluate changes in additional AAA-derived specialized metabolites in the transgenic leaves, we determined their fold change enhancement in comparison to controls (**Table 1**). The levels of a few of the identified phenylpropanoid metabolites increased in the *PheA*^∗^ lines, but most remained unaltered. The most significant effect detected was a dramatic increase in levels of rosmarinate, a conjugate between Phe-derived caffeate and Tyr-derived 3,4-dihydroxyphenylacetate (Petersen et al., 2009; Lee and Facchini, 2011), with 818- and 302-fold increase in *PheA^∗^* lines 8 and 19, respectively. Other significant enhancements were in the levels of benzoate (line 5), 4-hydroxyphenyl-β-glucopyranoside (lines 5, 8, 19), ferulate (line 5, 19), *t*-caffeate (lines 8, 19, 26), and *t*-coumarate (line 8).

### Effect of PheA^∗^ Accumulation in Flowers on the Levels of PPY, AAAs, and Their Downstream Non-volatile Metabolites

**FIGURE 3:**
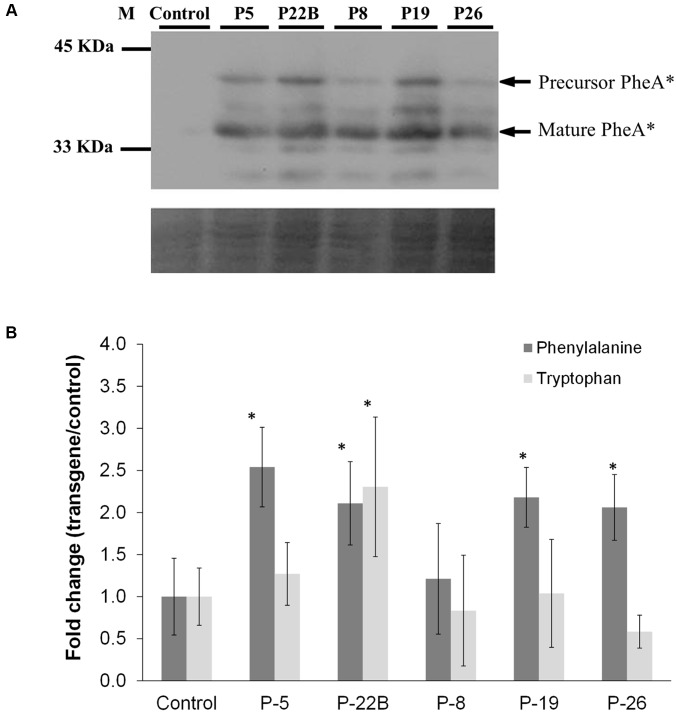
**Effect of PheA^∗^ protein abundance on the levels of the AAAs in *PheA^∗^* flowers. (A)** Accumulation of PheA^∗^ protein in the flower petals of the five transgenic plants. Immunoblot analysis was performed using anti-HA antibody (1:1000). Lower panel indicates similar protein loading by Amido-black staining. **(B)** Levels of the AAAs in flowers of control and the five *PheA*^∗^ transgenic lines. Results are presented as fold change in transgenic vs. control flowers (*n* = 3). Tyrosine was not identified. Asterisks indicate a statistically significant difference between the *PheA^∗^* lines and control, using *t*-test with *p* ≤ 0.05. Bars on top of the histograms indicate standard errors.

The abundance of the PheA^∗^ protein in the flower petals varied slightly between the five transgenic lines with a maximum of 2.5-fold (**Figure 3A**). As in the leaves, both the non-cleaved and the cleaved forms of the PheA^∗^ enzyme were detected. Several additional bands were also detected, in the high expressing lines, which may be a result of a degradation process.

The metabolic analysis was carried out on flowers, 1–2 days post-anthesis. Analysis of AAA levels showed mild but significant enhancement in Phe levels in four of the five *PheA^∗^* lines (**Figure 3B**). Tyr was not detected in the *PheA^∗^* and control petals. No significant change was observed in Trp levels, as expected, apart from a slight increase in line 22B.

Most of the non-volatile phenolic metabolites in the flower petals remained unchanged in the *PheA^∗^* lines, while some increased up to threefold (mainly *t*-caffeate, 4-caffeoyl-trans-quinate, and 4-hydroxyphenyl-β-glucopyranoside) (Supplementary Table S1A). Total anthocyanin levels did not increase in all five *PheA^∗^* lines, in comparison to control (results not shown). Analysis of changes in the flavonoids-anthocyanins pigments was conducted on *PheA^∗^* 26 line only (Supplementary Table S1B). The flavonols quercetin and kaempferol, intermediates in the flavonoid-anthocyanin pathway, were slightly reduced. No significant change was observed in the quantity or quality of the anthocyanidins, composing the flower pigments, delphinidin, petunidin, and malvidin (Supplementary Table S1B). This result is similar to the effect of overproduction of Phe in petunia by the bacterial feedback insensitive *AroG^∗^* enzyme (Oliva et al., 2015).

### Effect of PheA^∗^ Abundance on the Volatile Metabolic Profile of the Flower Petals

**FIGURE 4:**
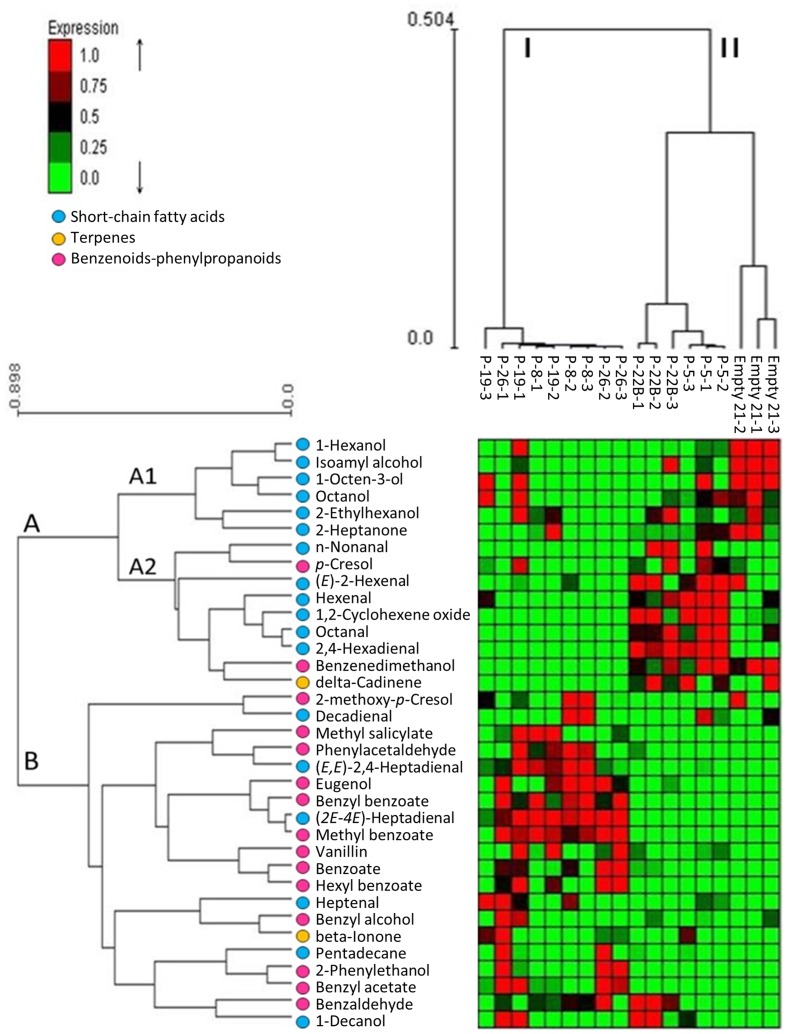
**Effect of PheA^∗^ transgene on the internal pools of volatile metabolites in the petals**. Results are presented by heat map hierarchical clustering of the volatiles. Analysis was performed in Expander (Ulitsky et al., 2010). Each column represents a sample from either *PheA^∗^* or control (Empty 21) flowers (*n* = 3). Each row represents a volatile. Values of volatiles’ internal pools were centered and scaled for the analysis and are presented by virtual colors as shown in the color key. Supplementary Table S2 presents the original absolute volatile internal pool levels. All volatiles were coded for their groups by key color. Benzenoid–phenylpropanoid volatiles marked by pink circles, short-chain fatty acids and terpenoids were marked by blue and yellow circle, respectively.

Changes in the internal pools of volatiles synthesized by petunia *PheA^∗^* petals were characterized by GC-MS SPME analysis. The identified volatiles included BP volatiles, short chain fatty acids and terpenoids (Supplementary Table S2). Their abundance in the different lines is presented in a hierarchical clustering heat-map and the characterized lines grouped into clusters I and II, and the identified volatiles in clusters A and B (**Figure 4**). *PheA^∗^* lines 8, 19, and 26 were clustered into one group (I) and *PheA^∗^* lines 5, 22B and the control line (named Empty 21) into another group (II) (**Figure 4** and Supplementary Table S2). Within group II, the control line was sub-clustered separately. Transgenic lines in cluster I (8, 19, 26) show a common pattern of high accumulation of volatiles mostly from group B. Lines grouped to cluster II (22B, 5, and control), showed enhanced levels of volatiles from group A2. Most of the BP volatiles were clustered into group B. The only two BP volatiles clustered in group A were *p*-cresol and benzene di-methanol, both possible catabolic products of phenolic compounds. The highest levels of BP volatiles were in lines 8, 19, and 26, in cluster I.

Other volatiles which were grouped into cluster A2 are terpenoids and short-chain fatty acids. The short-chain fatty acids accumulated to higher levels in cluster II (lines 5, 22B, and control). These volatiles are known to be formed as a result of membrane degradation and not due to biosynthesis processes.

**FIGURE 5:**
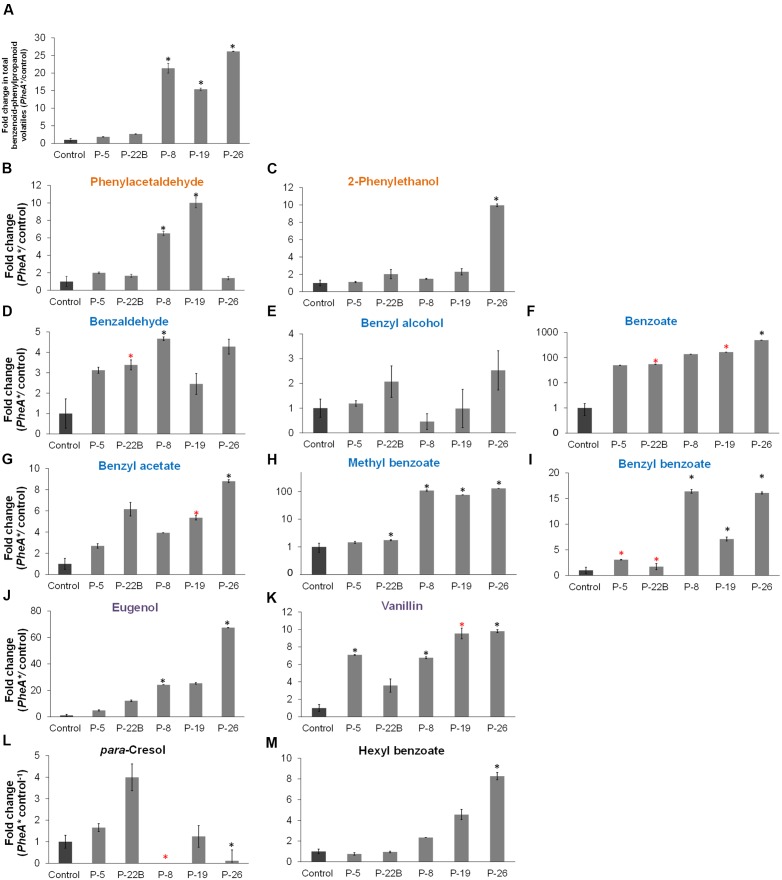
**Effect of the *PheA*^∗^ transgene on the Benzenoid–Phenylpropanoid volatiles in the petals. (A)** Fold enhancement of total BP volatiles in *PheA^∗^* lines in comparison to control (*n* = 3). **(B–M)** Fold enhancement of specific BP volatiles. Results are presented according to their biosynthesis in the three BP pathways in petunia petals. Volatiles names are colored according to their pathway as presented in **Figure 1** (orange, blue, and purple). Volatiles synthesized directly from Phe **(B,C)**, volatiles synthesized from cinnamate **(D–I)**, and volatiles synthesized in the pathway begins with ferulate **(J,K). (L,M)** BPs whose biosynthetic pathways have not been identified in petunia. Black and red asterisks indicate statistically significant differences (*p* ≤ 0.05 and *p* ≤ 0.1, respectively) between the *PheA^∗^* lines and the control, using ANOVA followed by Dunnett’s *post hoc* test after log transformation. Bars on top of the histograms indicate standard errors.

The five transgenic lines can be divided into two groups, based on the fold change increase in their total BP volatiles. The fold increase in total BP volatiles in lines 8, 19, and 26 was above 15-fold, while in lines 5 and 22B, the fold increase was statistically insignificant (**Figure 5A**). This is in accordance with the heat map results, clustering lines 8, 19, and 26 separately from lines 5 and 22B, and further apart from control (**Figure 4**). Lines 8, 19, and 26 had higher accumulation of total BP volatiles than lines 5 and 22B, even though PheA*^∗^* abundance was similar between the two groups (**Figures 3A**, **5A**).

The BP volatiles derived from all three pathways in *PheA^∗^* flowers (**Figure 1**) accumulated to significantly higher levels in comparison to control flowers, with a variability seen between the five transgenic lines (**Figure 5**). Line 26 accumulated the highest levels in most of the BP volatiles (**Figure 5A**). Two of the volatiles from the BP pathway 1 that initiates with phenylacetaldehyde (**Figure 1**) were enhanced (phenylacetaldehyde in lines 8 and 19, and 2-phenylethanol in line 26, with a 10-fold enhancement; **Figures 5B,C**). Five volatiles synthesized from the pathway 2 mediated by *t*-cinnamate increased in part of the transgenic lines, including benzaldehyde, benzoate, benzyl acetate, methyl benzoate, and benzyl benzoate (**Figures 5D–I**). The highest fold increase was in benzoate and methyl benzoate, with up to 100-fold increase in methyl benzoate and up to 500-fold increase in benzoate. Two volatiles synthesized from the pathway beginning with *p*-coumarate, eugenol and vanillin, increased up to 70- and 10-fold, respectively in part of the *PheA*^∗^ lines (**Figures 5J,K**). The levels of *p*-cresol and hexyl benzoate (**Figures 5K,L**) increased in part of the transgenic lines up to eightfold. Both metabolites have been identified previously in petunia flowers (Oliva et al., 2015) but their biosynthetic pathway is still not known.

## Discussion

Production of Phe *via* PPY is a well-known route in microorganisms (Zhang et al., 1998) but has only recently been elucidated in plants (Tzin et al., 2009, 2015; Yoo et al., 2013). Overexpressing the feedback insensitive bacterial bi-functional CM/PDT gene, *PheA^∗^*, directing carbon flux toward the PPY-Phe route, enabled further understanding of this route, and in particular the effect of increased PPY production on specialized metabolism both in the leaves and flowers.

The most significant effect of *PheA^∗^* overexpression in petunia, was the dramatic increase in their BP volatiles (**Figures 4**, **5**) from all three pathways present in petunia flowers (**Figure 1**). The level of total BPs in three of the five transgenic lines increased more than 25-fold in comparison to controls, with 500- and 100-fold increases in benzoate and methyl benzoate, respectively (**Figures 5F,H**). These results are particularly striking when we compare the metabolic profile of these *PheA*^∗^ flowers to flowers from of the same cultivar (*P. hybrida* V26) overexpressing the *AroG^∗^* gene (Oliva et al., 2015). *AroG^∗^* is a bacterial feedback insensitive enzyme with the activity of DAHPS (deoxy-arabino-heptulosonate 7-phosphate synthase), the first enzyme in the shikimate pathway (Tzin et al., 2012).

The enhancement in the levels of total BP volatiles in *PheA^∗^* petunia was similar to those of the *AroG^∗^* flowers, even though the increase in Phe levels in the *PheA^∗^* flowers (2- to 2.5-fold) was about 10-fold lower than in *AroG^∗^* flowers (**Figure 3B**, Oliva et al., 2015). Since we expect the BP production pathways to be identical in the transgenic *PheA^∗^* and *AroG^∗^* petunia flowers, we assume that the higher levels of Phe in *AroG^∗^* was due to a higher production rate of Phe in these flowers in comparison to *PheA^∗^*. The low increase in Phe levels in *PheA^∗^* flowers is in agreement with previous studies that have shown that most of the carbon flux in petunia is directed *via* the arogenate-Phe pathway and not *via* PPY (**Figure 1**; Maeda et al., 2010; Yoo et al., 2013). It is important to state that Phe levels are rate limiting in the production of BPs in petunia, since feeding of petunia cut flowers with external Phe caused an increase in internal BP pools to levels much higher than those in both *AroG^∗^* and *PheA^∗^* flowers (Oliva et al., 2015). Further proof for the importance of Phe levels for BP production is the fact that knockdown mutants of Phe-Tyr biosynthetic enzymes in petunia flowers reduced Phe levels and also caused a dramatic decrease in the levels of BP volatiles (Colquhoun et al., 2010; Maeda et al., 2010, 2011).

In addition to low Phe levels, the levels of non-volatile metabolites downstream of Phe and Tyr were also very mild in *PheA^∗^* flowers, in comparison to *AroG^∗^* flowers (Supplementary Table S1; Oliva et al., 2015). The equivalent levels of BP internal pools in *AroG^∗^* and *PheA^∗^* flowers, despite the differences in Phe and non-volatile phenylpropanoid levels, suggest that there may be an alternative pathway for direct synthesis of BPs from PPY. Several such pathways have been proposed, including direct synthesis of phenylacetaldehyde from PPY (Watanabe et al., 2002; Hirata et al., 2016) and possible direct synthesis of benzaldehyde from PPY (Orlova et al., 2006). Existence of such pathways in petunia may explain the dramatic increase in fragrant volatiles in *PheA^∗^* flowers, in contrast to the low accumulation of non-volatile metabolites downstream of Phe. Since feeding experiments with labeled PPY are problematic because PPY can converts spontaneously to benzaldehyde in the feeding solution (Orlova et al., 2006), *PheA^∗^* flowers are advantageous in studying the conversion of PPY to volatile BPs. In *PheA*^∗^, PPY is enhanced *in planta* in the relevant site of production inside the cells. Conversion of PPY to specified BPs may involve enzymes (Hirata et al., 2016), or perhaps, similar to what was suggested in lactic acid bacteria (Groot and de Bont, 1998), to occur spontaneously in the cells. Testing the metabolic effect of inhibiting PAL activity in *PheA^∗^* flowers may help resolve this issue. Increased accumulation of BPs in *PheA^∗^* flowers in which PAL activity is inhibited will be proof to direct production of BPs from PPY.

Similar to petunia, tomato *AroG^∗^* transformation caused a significant increase both in non-volatile and volatile BPs in the fruit (Tzin et al., 2013, 2015), while *PheA^∗^* transformation affected mainly the volatile metabolites (Tzin et al., 2015). However, unlike petunia flowers, the increase in volatiles in *PheA^∗^* tomato was significantly lower than that in *AroG^∗^* fruit. This suggests that BPs formation from PPY may differ between petunia flowers and tomato fruit.

**FIGURE 6:**
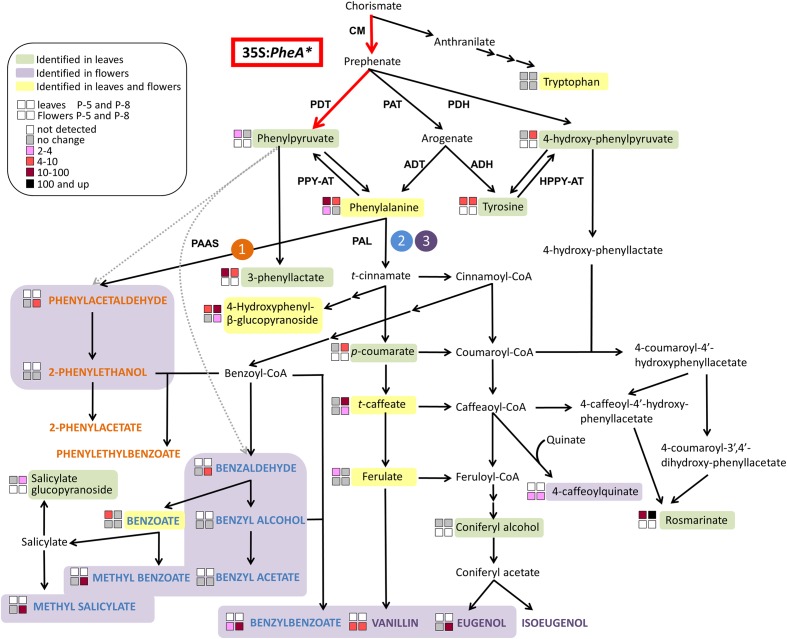
**A metabolic map describing the effect of overexpression of *PheA^∗^* on the metabolic profile of petunia flowers and leaves in the two transgenic lines 5 and 8**. The BP volatile metabolic pathways originating from Phe in petunia flowers are marked by orange **(1)**, blue **(2)**, and purple **(3)**. The volatile compounds are marked by colored bold capital letters. Enzymatic steps performed by the bi-functional feedback insensitive PheA^∗^ enzyme are marked in red. Enzymes are marked in bold capital letters. Multiple arrows mark several biochemical reactions. Dotted gray arrows mark reactions that have been shown in other organisms (production of phenylacetaldehyde from PPY in rose, and production of benzaldehyde from PPY in lactic acid bacteria or spontaneously) and may yet be revealed in petunia. A dashed line separates between primary to specialized metabolites. ADH, arogenate dehydrogenase; ADT, arogenate dehydratase; CM, chorismate mutase; HPPY-AT, hydroxyphenylpyruvate aminotransferase; PAAS, phenylacetaldehyde synthase; PAL, phenylalanine ammonia lyase; PAT, prephenate aminotransferases; PDH, prephenate dehydrogenase; PDT, prephenate dehydratase; PPY-AT, phenylpyruvate aminotransferase.

The five *PheA^∗^* transgenic lines are clearly divided into two groups (lines 5 and 22B in one group and lines 8, 19, and 26 in the second group) based both on their total BP values (**Figure 5A**) and on the hierarchical heat-map, clustering all volatiles (**Figure 4**). Interestingly, the metabolic analysis of petunia leaves from the five transgenic *PheA^∗^* lines, revealed a similar division between the lines, with lines 5 and 22B having a related pattern and differing from lines 8 and 19 (**Table 1**). Line 26 was unique, since based on the leaf metabolic profile it did not fit either of the two groups, but based on the flower metabolic profile line 26 clustered with lines 8 and 19 (**Table 1** and **Figure 4**). In an attempt to understand the possible differences between the two groups, we summarized the metabolic changes both in leaves and flowers in the two lines, 5 and 8, as representatives of the two groups (**Figure 6**).

The three metabolites along the AAA biosynthetic pathway that accumulated to higher levels in *PheA^∗^* leaves but were not identified in the flowers were PPY, Tyr, and 4-HPPY (**Figure 6**). The two lines, 5 and 8, differed already at this biosynthetic level, with increased PPY only in line 5 and increased 4-HPPY only in line 8. 3-Phenyllactate, synthesized from PPY, accumulated in the leaves of both lines, but to a higher degree in line 5. Tyr levels were elevated to similar levels in both lines. The most significant metabolic effect in petunia leaves due to *PheA^∗^* overexpression was accumulation of rosmarinate (**Figure 6**). Petunia leaves have been shown to accumulate high rosmarinate levels following cold stress (Pennycooke et al., 2005), and microbial attack (Szabo et al., 1999). Here we show that both lines accumulated rosmarinate, but there was a dramatic difference between them with a 10-fold increase in line 5 and an 800-fold increase in line 8, in comparison to the control (**Figure 6**).

The changes in the leaf metabolic profile of the five transgenic lines suggest that the group represented by line 8, has a strong carbon shift toward the synthesis of 4-HPPY and its downstream metabolite rosmarinate (**Figure 6**). Increased accumulation of 4-HPPY has also been suggested to occur in *Arabidopsis* PheA^∗^ plants, since these plants accumulate high levels of homogenisate, a metabolites produced from 4-HPPY (Tzin et al., 2009). Interestingly, the same group of transgenic lines, represented by line eight, accumulating high levels of rosmarinate in their leaves, also accumulated higher levels of BPs in their flowers (**Table 1** and **Figures 5**, **6**).

One possible explanation for the increase in 4-HPPY and rosmarinate in petunia leaves and 4-HPPY-derived metabolites in *Arabidopsis* leaves, is a “leak” of prephenate from the bifunctional enzyme PheA^∗^, which is then converted to 4-HPPY either directly or *via* arogenate and tyrosine (**Figure 6**). This may occur since PheA has been shown to have a stronger CM activity in comparison to PDT (Zhang et al., 1998), possibly resulting in more prephenate molecules for each PPY formed. Therefore, in case of increased PheA^∗^ activity in the group represented by line 8, more prephenate will be available for the synthesis of 4-HPPY.

A second possible explanation is based on a recent study characterizing the activity of PPY-AT. PPY-AT was shown to convert PPY to Phe, in a reaction coupled to the deamination of Tyr and formation of 4-HPPY and Phe (Yoo et al., 2013). Here too, increased activity of *PheA^∗^*, causing an increase in PPY synthesis, may result, due to the coupled reaction with tyrosine, in increased concentrations of 4-HPPY and Phe (**Figure 6**).

## Conclusion

Overproduction of *PheA^∗^* in petunia plants resulted in a dramatic increase both in the BP volatiles produced in flowers and in 4-HPPY-derived metabolites, in particular rosmarinate, in their leaves. The comparison between the *PheA^∗^* transgenic lines emphasized this dual metabolic result in petunia, since the group of *PheA^∗^* lines with a higher production of BPs was that with higher production of 4-HPPY and rosmarinate. Future detailed studies using labeled PPY or chorismate in *PheA^∗^* petunia may reveal the processes occurring in the flowers and leaves resulting in the metabolic profiles described, and in particular pathways for production of fragrant BPs directly from PPY.

## Author Contributions

MO and MO-S designed the experiments. MO, EB, and RO conducted the experiments. MO, MO-S, and EL interpreted and prepared the manuscript. AP and GG revised the manuscript. All authors have read and approved the manuscript for publication.

## Conflict of Interest Statement

The authors declare that the research was conducted in the absence of any commercial or financial relationships that could be construed as a potential conflict of interest.

## Footnotes

1 www.nist.gov

2 www.metaboanalyst.ca

## Supplementary Material

FIGURE S1 **Original Gel Describing the Effect of PheA^∗^ protein abundance on the AAAs and shikimate pathway intermediates in the leaves**. Accumulation of PheA^∗^ protein in the leaves of the five transgenic plants. Immunoblot analysis was performed using anti-HA antibody (1:1000). Lower panel indicates similar protein loading by Amido-black staining.

## Abbreviations

AAA: aromatic amino acids
BP: benzenoids–phenylpropanoids
PAL: phenylalanine ammonia lyase
Phe: phenylalanine
PPY: phenylpyruvate
Trp: tryptophan
Tyr: tyrosine.
