# Supplementary figures and images for: Phenylpyruvate Contributes to the Synthesis of Fragrant Benzenoid–Phenylpropanoids in Petunia × hybrida Flowers
Source: Front Plant Sci. 2017 May 12;8:769. doi: 10.3389/fpls.2017.00769 (PMC5427144; doi:10.3389/fpls.2017.00769)

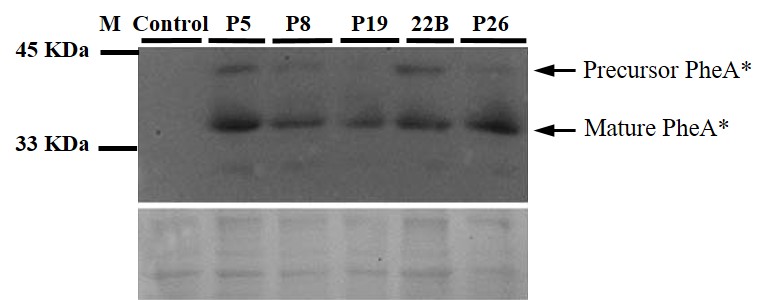

Supplement: FIGURE S1 — Original Gel Describing the Effect of PheA∗ protein abundance on the AAAs and shikimate pathway intermediates in the leaves. Accumulation of PheA∗ protein in the leaves of the five transgenic plants. Immunoblot analysis was performed using anti-HA antibody (1:1000). Lower panel indicates similar protein loading by Amido-black staining. [file Image_1.JPEG]
