# Supplementary material for: Phenylpyruvate Contributes to the Synthesis of Fragrant Benzenoid–Phenylpropanoids in Petunia × hybrida Flowers
Source: Front Plant Sci. 2017 May 12;8:769. doi: 10.3389/fpls.2017.00769 (PMC5427144; doi:10.3389/fpls.2017.00769)
Supplement: Supplementary file 2 [file Table_1.PDF]

Supplementary Material

Bypassing the Arogenate Pathway in *Petunia X Hybrida* Flowers Using A Mutated Feedback Insensitive Bacterial *PheA* Gene Results in Enhanced Levels of Fragrant Volatile Benzenoid-Phenylpropanoids

Moran Oliva, Einat Bar, Rinat Ovadia, Avichai Perl, Gad Galili, Efraim Lewinsohn, and Michal Oren-Shamir*

***Correspondence:** Michal Oren-Shamir: vhshamir@agri.gov.il

Table S1 Levels of soluble metabolites related to AAA in petals of *PheA** lines in comparison to control. The soluble fraction of petal metabolites was analyzed by (A) GC-MS following derivatization of the metabolites. (B) HPLC for flavonoids and anthocyanidins of *PheA**_26. Results are presented as fold change of transgene/control, (n=3). Statistical significance is marked by bold performed after log transformation of the data, analyzed by ANOVA followed by Dunnett's post-hoc (p<0.05).

**A- GC-MS**	**Control**	**P-5**	**P-22B**	**P-8**	**P-19**	**P-26**
Benzoate	1.00±0.05	1.23±0.13	0.93±0.42	0.40±0.64	0.55±0.32	1.63±0.11
4-caffeoyl-*t*- quinate	1.00±0.12	**2.52**±0.13	**3.11**±0.17	**3.01**±0.30	**2.70**±0.12	**3.17**±0.06
t-caffeate	1.00±0.19	2.26±0.16	**3.10**±0.26	**2.82**±0.22	2.05±0.05	**2.94**±0.30
cis ferulate	1.00±0.02	1.58±0.12	2.03±0.19	2.60±0.64	1.88±0.23	2.67±0.29
Homovanillate	1.00±0.13	0.68±0.04	0.82±0.63	0.39±0.24	0.81±0.63	1.77±0.25
4-Hydroxy-benzoate	1.00±0.11	1.11±0.24	0.95±0.22	1.06±0.55	1.06±0.30	1.36±0.19
5-Hydroxy-tryptophan	1.00±0.04	0.91±0.09	0.88±0.16	0.96±0.19	0.85±0.18	0.74±0.07
4-Hydroxyphenyl-β-glucopyranoside	1.00±0.21	1.27±0.28	1.54±0.35	**2.01**±0.32	1.16±0.41	1.51±0.24
Phenethylamine	1.00±0.10	1.50±0.28	0.71±0.24	0.81±0.15	0.68±0.27	1.20±0.41
Salicin	1.00±0.03	1.21±0.11	0.97±0.13	0.90±0.44	1.00±0.39	0.94±0.19
Tyrosol	1.00±0.13	0.67±0.03	0.81±0.62	0.38±0.25	0.80±0.62	1.74±0.24

**B- HPLC**	**Control**	**P-5**	**P-22B**	**P-8**	**P-19**	**P-26**
Quercetin	1.00 ±0.04	-	-	-	-	**0.70**±0.04
Kaempferol	1.00 ±0.04	-	-	-	-	**0.87**±0.01
Delphinidin	1.00 ±0.37	-	-	-	-	1.02±0.04
Petunidin	1.00 ±0.17	-	-	-	-	0.96±0.05
Malvidin	1.00 ±0.08	-	-	-	-	1.05±0.04

Table S2 Volatiles internal pools synthesized by the transgenic *PheA** petals analyzed by SPME based GC-MS. Volatiles were grouped into FA= fatty acid, BPV= benzenoids-phenylpropanoid volatiles and Terpenoids. Averages±SE results of absolute levels (ng/mg FW) are presented. Statistically significant molecules are marked by bold, were analyzed by ANOVA followed by Dunnett's post-hoc assay after log transformation of the data with, $p \leq 0.05$.

Compound name	Group	Control	P-5	P-22B	P-8	P-19	P-26
1-Decanol	FA	0.05±0.01	0.14±0.04	**0.30**±0.07	0.10±0.00	**0.19**±0.06	0.15±0.06
1-Hexanol	FA	13.47±1.03	**6.32**±0.87	**4.35**±1.05	**3.36**±0.35	6.64±2.44	**3.54**±1.04
1-Octen-3-ol	FA	0.03±0.00	0.02±0.00	**0.02**±0.00	**0.02**±0.00	0.02±0.01	**0.02**±0.00
2-4-Hexadienal	FA	0.08±0.04	**0.43**±0.06	**0.33**±0.02	**0.00**±0.00	**0.00**±0.00	**0.00**±0.00
2E-4E-Heptadienal-	FA	0.01±0.00	**0.03**±0.01	**0.03**±0.00	**0.62**±0.01	**0.58**±0.09	**0.65**±0.06
2E-Hexenal	FA	3.93±1.39	4.82±0.18	4.39±0.69	3.54±0.35	3.41±0.30	2.04±0.18
2-Ethylhexanol	FA	0.07±0.01	0.05±0.01	0.08±0.02	0.05±0.01	0.06±0.03	**0.00**±0.00
2-Heptanone	FA	1.31±0.07	1.23±0.02	1.20±0.00	1.18±0.01	1.20±0.05	1.01±0.03
2-methoxy-*para*-Cresol	BPV	0.30±0.21	0.16±0.02	0.10±0.00	0.64±0.27	0.36±0.04	0.18±0.06
2-Phenylethanol	BPV	0.77±0.25	0.86±0.06	1.09±0.28	1.04±0.06	1.76±0.63	**7.65**±1.19
Benzaldehyde	BPV	0.86±0.62	2.69±0.40	**5.92**±0.63	**4.21**±0.10	2.11±1.08	3.69±1.35
Benzene di-methanol	BPV	0.08±0.01	0.09±**0.01**	0.07±0.01	0.01±0.01	0.02±0.01	0.03±0.01
Benzoate	BPV	0.00±0.00	0.24±0.09	**0.70**±0.21	0.67±0.38	**0.81**±0.34	**2.41**±0.54
Benzyl acetate	BPV	0.01±0.00	0.03±0.01	0.03±0.00	0.02±0.01	**0.05**±0.01	**0.08**±0.01
Benzyl alcohol	BPV	2.09±0.77	2.49±0.28	2.38±0.52	1.60±0.37	2.07±1.60	5.30±4.21
Benzyl benzoate	BPV	0.48±0.28	**1.46**±0.07	**1.46**±0.41	**7.09**±0.41	**3.39**±1.30	**7.67**±1.53
Beta-Ionone	Terpene	0.01±0.00	0.02±0.01	0.02±0.00	0.01±0.00	0.03±0.01	**0.06**±0.03
Decadienal	FA	0.05±0.05	0.10±0.05	0.01±0.00	0.26±0.14	0.01±0.00	0.01±0.00
delta-Cadinene	Terpene	0.05±0.04	0.07±0.02	0.07±0.02	0.01±0.01	**0.00**±0.00	**0.00**±0.00
E-E-2 2-4-Heptadienal	FA	0.01±0.01	0.07±0.02	0.05±0.00	**0.17**±0.01	**0.15**±0.03	**0.11**±0.00
Eugenol	BPV	0.00±0.00	0.02±0.01	0.04±0.03	**0.20**±0.07	0.08±0.05	**0.22**±0.04
Heptenal	FA	0.07±0.03	0.15±0.01	0.10±0.01	0.14±0.04	0.24±0.09	0.16±0.06
Hexanal	FA	4.61±2.65	13.34±1.20	9.37±0.61	5.04±0.03	7.32±1.08	3.61±0.96
Hexyl benzoate	Terpene	0.02±0.00	0.01±0.00	0.02±0.00	0.03±0.00	0.07±0.03	**0.13**±0.05
Isoamylalcohol	FA	1.27±0.04	**0.52**±0.09	0.64±0.19	**0.19**±0.01	**0.35**±0.17	**0.20**±0.04
Methyl benzoate	BPV	1.00±0.36	1.47±0.18	**2.54**±0.48	**96.81**±7.05	**75.93**±22.57	**130.05**±13.38
Methyl salicylate	BPV	0.00±0.00	0.00±0.00	0.00±0.00	**2.03**±2.01	**4.62**±2.44	**1.36**±0.69
n-Nonanal	FA	0.00±0.00	0.00±0.00	0.01±0.00	0.00±0.00	0.00±0.00	0.00±0.00
Octanal	FA	0.03±0.01	0.08±0.02	0.06±0.01	0.00±0.00	0.00±0.00	0.00±0.00
Octanol	FA	2.12±0.28	1.82±0.14	1.30±0.20	0.72±0.02	2.13±0.65	**0.70**±0.13
p-Cresol	BPV	0.22±0.07	0.37±0.07	0.58±0.31	**0.00**±0.00	0.28±0.14	**0.03**±0.01
Pentadecane	FA	0.02±0.00	0.04±0.00	0.03±0.00	0.00±0.00	0.02±0.01	**0.10**±0.06
Phenylacetaldehyde	BPV	0.19±0.11	0.37±0.03	0.69±0.22	**1.69**±0.27	**1.87**±1.08	0.26±0.05
Vanillin	BPV	0.18±0.07	**1.30**±0.06	0.95±0.28	**1.03**0.12	**1.74**±0.80	**1.79**±0.90
